# Retraction: Huang et al. The Design, Fabrication and Characterization of Grating Couplers for SiGe Photonic Integration Employing a Reflective Back Mirror. *Nanomaterials* 2022, *12*, 3789

**DOI:** 10.3390/nano13101626

**Published:** 2023-05-12

**Authors:** Qiang Huang, Yi Zhang, Jie Tang, Junqiang Sun

**Affiliations:** 1 Hunan Provincial Key Laboratory of Grids Operation and Control on Multi-Power Sources Area, School of Electrical Engineering, Shaoyang University, Shaoyang 422000, China; 2 Wuhan National Laboratory for Optoelectronics, School of Optical and Electronic Information, Huazhong University of Science and Technology, Wuhan 430074, China

The Journal retracts the article “The Design, Fabrication and Characterization of Grating Couplers for SiGe Photonic Integration Employing a Reflective Back Mirror” [1].

Following publication, the authors of [1] contacted the Editorial Office regarding duplicate publications and a retraction request. The authors informed the Editorial Office that the same study had been published previously in *Laser and Optoelectronics Progress* [2], in Chinese. Adhering to our procedure, an investigation was conducted that confirmed the overlap included identical figures, as well as the analysis of the samples and data interpretation. The Editorial Office confirmed that re-publication in English was conducted without the appropriate authorization from the Publisher of *Laser and Optoelectronics Progress*. The two manuscripts were also found to have minor differences in authorship. The article in *Nanomaterials* was published later than the one in *Laser and Optoelectronics Progress*, so following the Committee on Publication Ethics (COPE) guidelines on duplicated publication and in order to preserve the integrity of the scientific record, this article is therefore retracted.

This retraction was approved by the Editor in Chief of the journal *Nanomaterials*.

The authors agreed to this retraction.
